# Diversity of warning signal and social interaction influences the evolution of imperfect mimicry

**DOI:** 10.1002/ece3.4272

**Published:** 2018-07-03

**Authors:** Renan Janke Bosque, J. P. Lawrence, Richard Buchholz, Guarino R. Colli, Jessica Heppard, Brice Noonan

**Affiliations:** ^1^ The University of Mississippi University Mississippi; ^2^ Universidade de Brasília Brasília Brazil

**Keywords:** aposematism, Batesian, generalization, mimicry, Mullerian, social learning, warning signal

## Abstract

Mimicry, the resemblance of one species by another, is a complex phenomenon where the mimic (Batesian mimicry) or the model and the mimic (Mullerian mimicry) gain an advantage from this phenotypic convergence. Despite the expectation that mimics should closely resemble their models, many mimetic species appear to be poor mimics. This is particularly apparent in some systems in which there are multiple available models. However, the influence of model pattern diversity on the evolution of mimetic systems remains poorly understood. We tested whether the number of model patterns a predator learns to associate with a negative consequence affects their willingness to try imperfect, novel patterns. We exposed week‐old chickens to coral snake (*Micrurus*) color patterns representative of three South American areas that differ in model pattern richness, and then tested their response to the putative imperfect mimetic pattern of a widespread species of harmless colubrid snake (*Oxyrhopus rhombifer*) in different social contexts. Our results indicate that chicks have a great hesitation to attack when individually exposed to high model pattern diversity and a greater hesitation to attack when exposed as a group to low model pattern diversity. Individuals with a fast growth trajectory (measured by morphological traits) were also less reluctant to attack. We suggest that the evolution of new patterns could be favored by social learning in areas of low pattern diversity, while individual learning can reduce predation pressure on recently evolved mimics in areas of high model diversity. Our results could aid the development of ecological predictions about the evolution of imperfect mimicry and mimicry in general.

## 1 INTRODUCTION

Mimicry is an evolutionary strategy often employed by organisms to escape predation. Mimetic phenotypes can generally be classified as either camouflage/masquerade, for example, insects mimicking leaves (Skelhorn & Ruxton, 2010) or warning, that is, co‐opting the signal of a defended prey species (Ruxton, Sherratt, & Speed, 2004). Color combinations including red, yellow, white, and black are broadly used as warning signals in many defended taxa, such as Hymenoptera (Hines & Williams, 2012), Coleoptera (Bocak & Yagi, 2010), Lepidoptera (Jiggins, Mallarino, Willmott, & Bermingham, 2006), Lissamphibia (Kraemer & Adams, 2014; Symula, Schulte, & Summers, 2001), and Squamata (Campbell & Lamar, 2004). These warning colors can elicit aversion in a wide variety of visually oriented predators (Ruxton et al., 2004). The aversion of conspicuous prey can even be socially transmitted (Thorogood, Kokko, & Mappes, 2017), reducing the predation pressure on newly evolved signals. Aversion can also be affected by individual variation in personality (Exnerová, Svádová, Fučíková, Drent, & Štys, 2010), which can be genetically inherited (Drent, Oers, & Noordwijk, 2003) and be accompanied by differences in morphological and physiological traits (Goerlich, Nätt, Elfwing, Macdonald, & Jensen, 2012). Whether this aversion is innate, self‐learned, or socially transmitted, warning signals are known to have a strong influence on how a predatory animal will explore and interact with prey (Aronsson & Gamberale‐Stille, 2012; Ham, Ihalainen, Lindstrom, & Mappes, 2006; Lindstrom, Alatalo, & Mappes, 1999; Rowe & Guilford, 2000).

**Figure 1:**
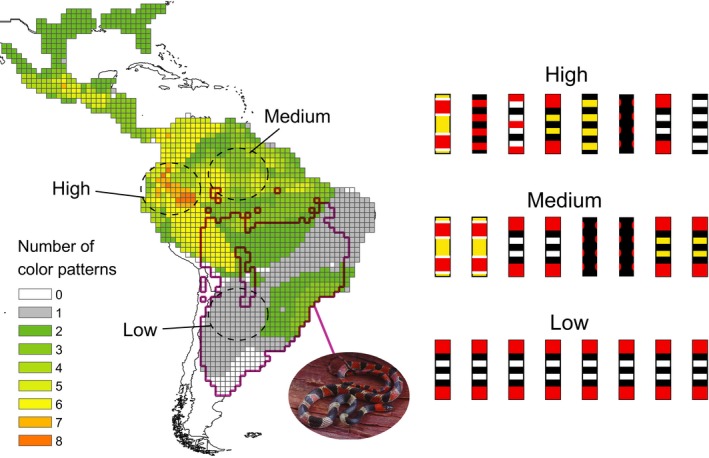
Map with one‐degree cells showing *Micrurus* color pattern richness. To the right are patterns used in the exposure phase. In pink the distribution of *Oxyrhopus rhombifer*. Map based on data from Bosque et al., 2016

At the community level, Batesian mimicry, where an undefended mimic benefits from a resemblance to a harmful model, is perhaps the most evolutionarily complex mimicry system (Bates, 1862; Ruxton et al., 2004). Multiple predator species may co‐occur with both multiple defended and multiple undefended prey species that employ a variety of warning colors and patterning, and the dimensionality of these components of the mimicry system can vary geographically. For example, New World coral snakes (*Micrurus*) and their mimics of the genus *Oxyrhopus* exhibit many combinations of model species number, mimic species number, pattern and coloration diversity (Figure 1), and extent of overlap between mimics and models (Bosque, Noonan, & Colli, 2016; Campbell & Lamar, 2004; Roze, 1996). Species of *Micrurus* transmit a clear warning signal to potential predators through varying combinations of contrasting red, black, yellow, and white rings (Brodie, 1993; Brodie & Janzen, 1995; Smith, 1976). These same colors are also used by harmless snakes, with varying fidelity in color and pattern to *Micrurus* models, making this one of most remarkable examples of mimetic interaction (Savage & Slowinski, 1992).

Regional variation in the warning coloration of mimics could occur simply because different predators may interpret mimic‐model resemblance using different sensory cues or cue components (Aubier & Sherratt, 2015; Pekar, Jarab, Fromhage, & Herberstein, 2011). Further, different populations of a mimetic species may occur in areas with different predators, with local color variants emerging by predation pressure. Nonetheless, even within a single predator species, individual experience with model pattern richness (i.e., the number of different prey patterns) by direct contact or via social observation may also directly affect the evolution of mimetic lineages.

A particularly vexing problem in the macroevolutionary study of mimicry complexes that might benefit from a deeper understanding of predator learning is that, despite a presumed selective pressure to attain perfect resemblance with their models, imperfect mimics are not uncommon in nature. The reasons for the maintenance of imperfect mimicry are still unclear but several authors have suggested plausible explanations (Kazemi, Gamberale‐Stille, Tullberg, & Leimar, 2014; Kikuchi & Pfennig, 2013). One explanation focuses on the selective pressures on the mimic when many models exist in the same area. When multiple models are present within a mimic's geographic distribution, mimics may be selected by predators to either resemble only one model or, if the models are not sympatric with each other, the mimics can adopt an intermediate phenotype (Edmunds, 2000; Sherratt, 2002). If just one model is present, selection is expected to drive mimics toward signal identity with the defended model (Ruxton et al., 2004). However, if several sympatric, defended models vary in phenotype, predators in this area may be conservative in the avoidance of harmless species with similar warning signals, even if mimicry of the defended models is inexact (Edmunds, 2000). Experimental evidence demonstrates that predators indeed generalize a bad experience with one prey species to others (Hotová Svádová, Exnerová, Kopečková, & Štys, 2013).

Model diversity may also drive generalization to novel patterns that are not even found in models (Ham et al., 2006; Kikuchi & Pfennig, 2013). Historically, avoidance of novel prey has been attributed to innate neophobia; the avoidance of a previously unencountered signal simply because it is new/unusual (Greenberg & Mettke‐Hofmann, 2001). Because neophobia may disappear with exposure experience, the generalization and neophobia hypotheses for explaining novel mimic‐like patterns make opposite predictions about the outcome of predator learning as the number of models increases. More models provide predators more cues from which to generalize, making them cautious about new prey patterns, but also increase the familiarity with novelty, thus fostering less neophobia toward it.

Previous researchers have demonstrated generalization of coral snake warning patterns by free‐ranging avian predators. In these studies, the birds avoided a mimetic morph with a pattern that differed from the local model but with the same colors (Brodie & Janzen, 1995; Kikuchi & Pfennig, 2010). To investigate the evolution of more complex systems with multiple models and imperfect mimics, we tested whether the number of models that an avian predator experiences affects the breadth of its avoidance generalization to a novel pattern. In this study, a “novel pattern” is also an imperfect mimic, a pattern not seen previously by the subject, and yet incorporating features (colors and shapes) shared with the aposematic models. We also exposed chickens to different contexts using social and individual exposure as these may affect learned responses to distasteful prey (Thorogood et al., 2017). In order to understand how differences in individual development of chicks could impact their willingness to sample imperfect mimics, we investigated morphological traits that may reveal ontogenetic growth trade‐offs between general investment in somatic growth (mass, tarsus and body condition) and organ‐specific development associated with immune preparedness (spleen mass) and sexual maturation (directional testis asymmetry). The spleen is an important immune organ in birds, the size of which reflects immune activity and possibly immunocompetence (John, 1994). As in most bird taxa, the left testis is usually larger in mature phasianid birds such as the chicken (Calhim & Montgomerie, 2015) and thus chicks with greater asymmetry in this direction can be assumed to be on a more rapid trajectory toward the adult form. Directional asymmetry in adult testis size has been associated with male sexual ornamentation and mate quality in some birds (Møller, 1994). We predicted that chicks who invest more in organ maturation would be more motivated to feed and thus less likely to avoid a novel food item, despite having learned previously that similar cues were aposematic.

## 2 METHODS

### 2.1 Study subjects and housing

As model predators we used approximately 10‐day‐old, male domestic chickens (*Gallus gallus domesticus*). The capacity of chickens to discriminate between two objects based on their wavelength is comparable to several bird species (Hart, 2001), which reinforces the adequacy of the species selected as model predator. Birds are commonly used as model predators in warning coloration experiments because their color vision is well documented, and they are known to be the main predators of snakes, including coral snakes (Buasso, Leynaud, & Cruz, 2006; Hinman et al., 1997; Kikuchi & Pfennig, 2010). Commercial chick feed (corn‐meal) was provided ad libitum except for the 60 min immediately prior to exposure and testing sessions, so that the chicks were motivated to “attack.” Housing and testing conditions were approved by the University of Mississippi Institutional Animal Care and Use Committee (#15‐009). To replicate the snake patterns found in nature, we painted Wild Harvest^™^ tube feeders with brown spray paint to represent brown snakes and wrapped experimental feeders with colored electrical tape to represent the coral snake color pattern(s) present in three regions of South America (Figure 1 and Supporting Information Figure S1) (Bosque et al., 2016). We filled the aposematic (henceforth, we use aposematic and warning signal interchangeably) feeders with chick feed that was previously sprayed with 10% chloroquine solution, making the feed distasteful but not harmful (Lindstrom, Alatalo, & Mappes, 1997; Ruxton et al., 2004); brown feeders had normal chick feed. These feeders were not meant to be exact replicas of coral snakes, but simply represent a variety of patterns from which the chicks had to learn. To simulate natural encounters with aposematic prey, we used two different approaches: group exposure and individual exposure. Using these two approaches, we could not only identify how pattern richness affected generalization to a new pattern but also the effect of social exposure versus individual exposure.

### 2.2 Group exposure

Chicks were housed in three groups of 43 in poultry brooder cages during exposure to aposematic feeders. Each exposure group experienced only one of the pattern richness treatment levels (Figure 1): highest color pattern richness—H (8 patterns), intermediate color pattern richness—M (4 patterns), or low color pattern richness—L (1 pattern).

In addition to regular (trough‐style) chick feeders, chicks were exposed to brown feeders for 8 hr per day during the first 4 days. On the 5th day, 16 bird feeders (8 brown and 8 aposematic) were positioned randomly along the perimeter of each enclosure for a 10 min exposure session. The feed in each feeder was weighed before and after each exposure session. This procedure was repeated an additional five times over 2 days. A final (6th) exposure session before testing lasted 1 hr, to ensure that chicks were completely avoiding the aposematic feeders. Notably, our group exposure training procedure allows for social learning (Slagsvold & Wiebe, 2011) as the chicks in the same cage may learn from each other's negative reaction to the feed in aposematic feeders. The learned aversion from conspecifics is still a theme that deserves investigation as contrasting results have been reported (Sherwin, Heyes, & Nicol, 2002; Thorogood et al., 2017).

### 2.3 Group testing

After the conclusion of group exposure, we individually tested chicks for their reaction to a feeder featuring either the imperfect mimetic pattern of the false coral snake (*Oxyrhopus rhombifer*) or a brown feeder. The testing arena consisted of a 60 cm × 60 cm wood box containing a small wire cage with two chick companions to prevent isolation stress of the test chick. Each chick was tested only once. Despite a broad geographic distribution, overlapping with many species of *Micrurus*,* Oxyrhopus rhombifer* has a tricolor pattern with black saddles bordered by white on a red dorsum (Figure 1), a pattern not found in any *Micrurus* species. A previous study using plasticine replicas has demonstrated that the *Oxyrhopus rhombifer* phenotype does provide protection against free‐range predators (Buasso et al., 2006), but the mechanisms of avoidance are still poorly understood.

We recorded the reaction to feeder exposure as the hesitation time (time until the first peck). Each trial lasted up to five minutes or until the first attack (peck). If we did not observe any attack after five min, we stopped the trial. Before each trial, we offered small pieces of dry mealworm (*Tenebrio molitor*) to ensure that chicks were hungry and willing to attack. All trials were recorded using a digital camera (videos available upon request).

### 2.4 Individual exposure

In order to explore the impact of individual exposure to different model community diversity we deprived 27 chicks of food for one hour. We then individually exposed 14 chicks to high color pattern richness (Figure 1)—H (8 patterns) and 13 chicks to low color pattern richness—L (1 pattern). Eight additional individuals were used as buddy chicks. The exposure (training) and testing arena consisted of a cardboard box 38 cm × 30 cm with two buddy chicks inside a small wire cage. In each treatment, we started by presenting one brown feeder for up to 2 min. Starting after the first peck, we allowed them to eat for a cumulative time of 10 s to prevent satiation. After that, we removed the brown feeder and presented a random aposematic feeder for up to 2 min. If the chick pecked the food, we allowed it to eat for up to a cumulative total of 10 s and then we removed the aposematic feeder. We repeated this procedure until all the 16 feeders were presented according to each subject's treatment group (H: 16 feeders with 8 different aposematic patterns; L: 16 feeders with 1 aposematic pattern—Supporting Information Figure S1) and recorded the hesitation time, that is, time until the first peck. We did not record the quantity of feed eaten by chicks during individual training.

### 2.5 Individual testing

After the exposure described above, we presented a feeder with an imperfect mimic (i.e., *Oxyrhopus rhombifer*) pattern alongside a brown feeder in the testing arena. The arrangement (left or right) of the feeders was randomized to avoid lateralization bias. We recorded the hesitation time and first feeder choice. To evaluate whether morphological characteristics could explain individual variation in hesitation time, we took the following postmortem measures of each chick at the end of the experiment: tarsus length, body mass, directional testes length asymmetry, spleen mass and body condition. The entire length of each testis was measured, unless the organ was not fully differentiated, in which case only the length of portion consisting of white (as opposed to purple‐red) tissue was measured. Directional testis asymmetry was calculated as (left length–right length). Body condition was calculated as mass/tarsus length (Brown, 1996).

### 2.6 Statistical analysis

We fitted Cox proportional hazards models to assess the dependency of hesitation time on predictor variables, using the *survival* package (Therneau, 2015) in R (R Core Team 2017). Survival analysis models the time (i.e., survival time) it takes for a given event to occur and the factors that affect it (Moore, 2016). For the group testing, we modeled hesitation time as a function of pattern richness exposure (H, M, or L), feeder type (aposematic or brown), and their interaction. For the individual testing, we modeled hesitation time as a function of pattern richness exposure (high or low), feeder type (aposematic or brown), their interaction, and the postmortem morphological variables (tarsus length, body mass, testis length asymmetry, spleen mass and body condition). We used stepwise model selection based on the Akaike information criterion (AIC) to assess predictor importance. For each model we checked (a) the proportional hazards assumption by examination of scaled Schoenfeld residuals using the *cox.zph* function of package *survival*; (b) the nonlinearity assumption using Martingale residuals; and (c) the presence of influential observations using case deletion residuals (dfbetas) (Moore, 2016). In all cases, we found no violation of assumptions or any influential observation. When needed, we performed pairwise comparisons of treatments using the log‐rank test as implemented by the function *pairwise_survdiff* in package *survminer* (Kassambara, Kosinski, Biecek, & Fabian, 2018), adjusting *p*‐values with the Benjamini–Hochberg's method (Benjamini & Yosef, 1995).

## 3 RESULTS

### 3.1 Group exposure

**Figure 2:**
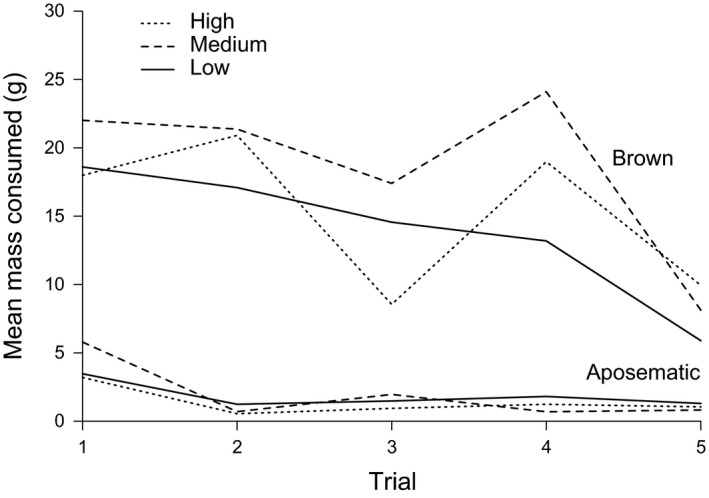
Bird food mass eaten by chickens after 10 min (rounds 1–5) of exposure. Top lines show feeders with brown coloration. Bottom lines show aposematic feeders (*Micrurus* patterns). High: eight aposematic patterns; medium: four aposematic patterns; low: one aposematic pattern

Across the first five exposure sessions, mean consumption of feed from the aposematic feeders was lower (H: 1.40 ± 1.44 g; M: 1.99 ± 2.68 g; L: 1.85 ± 3.13 g) than from the brown feeders (H: 15.27 ± 8.42 g; M: 18.60 ± 8.36 g; L: 14.20 ± 7.43 g). This pattern was found for all three cages in all exposure sessions (Figure 2). The last session (#6) demonstrated that the chicks were avoiding the aposematic patterns: brown feeders were nearly empty, whereas aposematic feeders were largely avoided (average of food left inside the feeders during the #6 session H: aposematic: 77.4%, brown: 17.10%; M: aposematic: 85.67%, brown: 8.06%; L: aposematic: 84.11%, brown: 27.22%).

**Figure 3:**
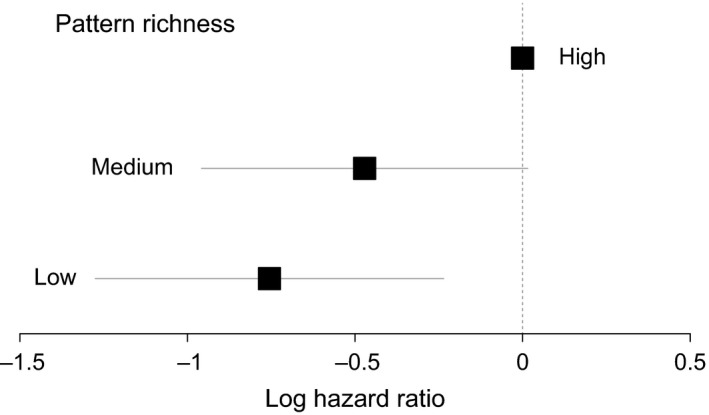
Survival analysis modeling hesitation time for chicks exposed as a group to different coral snake pattern richness to peck at feeders painted with nonaposematic (brown) or aposematic‐imperfect patterns as a function of pattern richness. Graphs depict log hazard ratios estimated by a Cox proportional hazards model having high color pattern richness as reference compared to log hazard ratio of medium and low pattern richness; horizontal bar represents 95% confidence interval

During the testing, we recorded a wide range of attack latencies from 1 s to 228 s. In 16 trials chicks never attacked the feeder, and thus their trials were terminated at 5 min, and these data were right‐censored in our survival analysis. The final model derived from analysis of group exposure contained only one predictor: pattern richness exposure (*r* ^2^ = 0.074, Wald test = 8.48, *df* = 2, *p *=* *0.014). Chicks exposed to low pattern richness had 0.47 times less risk of pecking the novel aposematic feeder than chicks in the high pattern richness treatment (log hazard ratio for low pattern richness exposure = −0.755, *Z *=* *−2.848, *p *=* *0.004, Figure 3, Supporting Information Figure S2). The birds in the medium richness treatment showed only a marginal difference from the high pattern richness group in the risk of pecking the feeder (log hazard ratio for medium pattern richness exposure = −0.47, *Z *=* *−1.898, *p *=* *0.058, Figure 3 Supporting Information Figure S2). Hesitation time differed only between low and high pattern richness, based on pairwise comparisons (Benjamin–Hochberg adjustment; high–low: *p *=* *0.001; high‐medium: *p *=* *0.081; low‐medium: *p *=* *0.293).

### 3.2 Individual exposure

**Figure 4:**
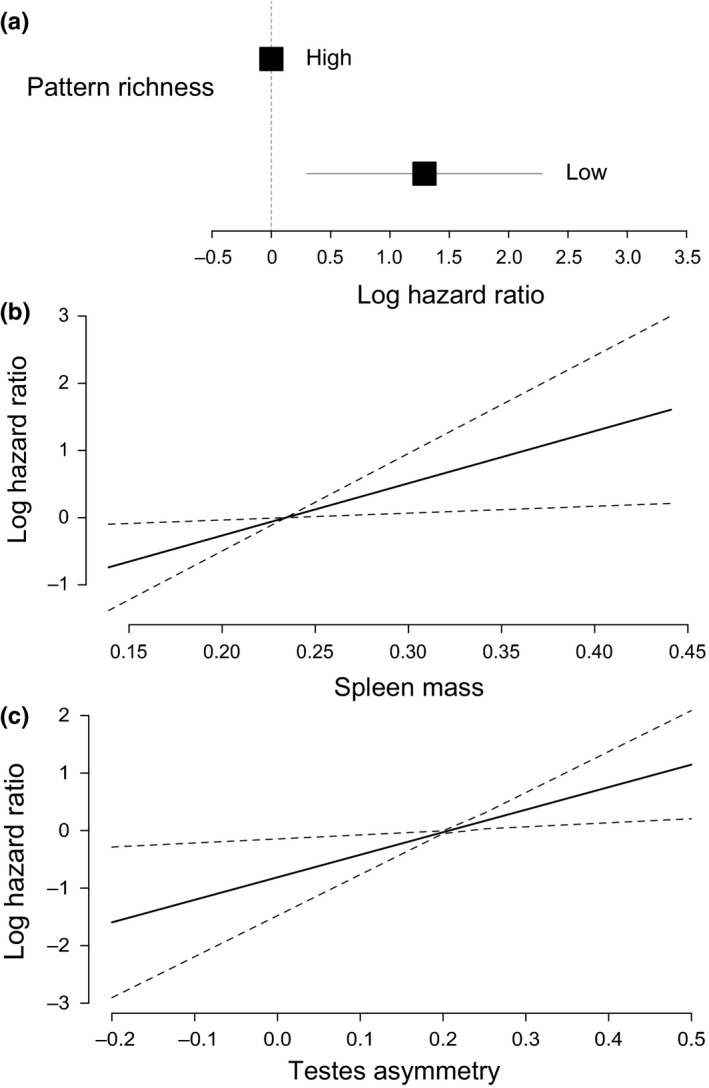
Survival analysis modeling hesitation time for chicks individually exposed to different coral snake pattern richness to peck on feeders painted with nonaposematic (brown) and aposematic‐imperfect patterns as a function of pattern richness, spleen mass, and testes asymmetry. Graphs depict log hazard ratios estimated by a Cox proportional hazards model as a function of the three predictors. (a) Log hazard ratio reference (high color pattern richness) compared to log hazard ratio of low pattern richness; horizontal bar represents 95% confidence interval. (b) Linear fit of the log hazard ratio as a function of spleen mass; dashed line represents 95% confidence interval. (c) Linear fit of the log hazard ratio as a function of testes asymmetry; dashed line represents 95% confidence interval

**Figure 5:**
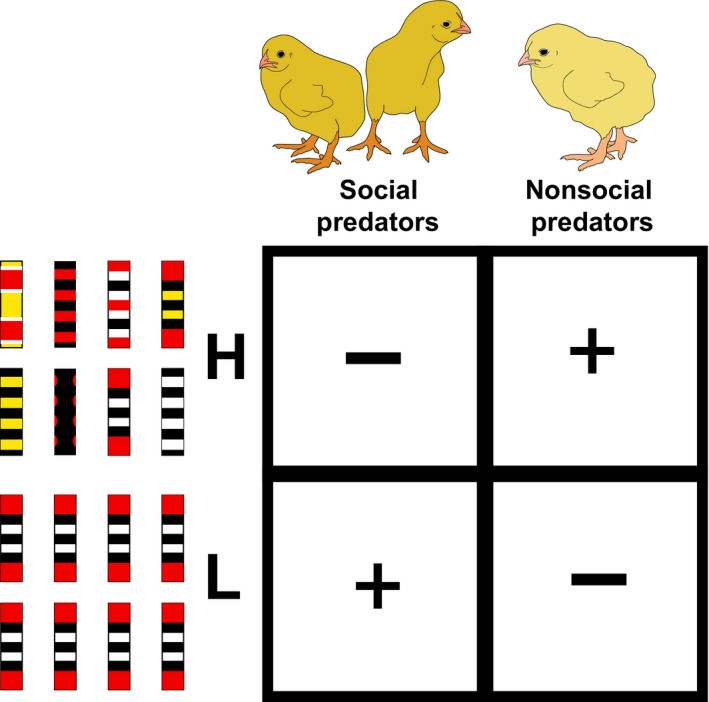
Diagram showing the effect of social and nonsocial predators on the evolution of mimicry/color pattern diversity. In areas of high model color diversity (H), new color patterns can be favored (+) by reduced predation pressure as a result of higher attack hesitation of nonsocial predators and disfavored (−) by lower attack hesitation of social predators. In areas of low pattern diversity (L), new color patterns can be favored (+) by reduced predation pressure as a result of higher attack hesitation of social predators and disfavored (−) by lower attack hesitation of nonsocial predators

When presented individually, feeder pattern (brown or aposematic imperfect) was not a part of our final model, showing that chicks had no preference for feeder type. The final model contained only three predictors: pattern richness exposure (high vs. low), spleen mass and directional testes asymmetry (*r* ^2^ = 0.445, Wald test = 13.3, *df* = 3, *p *=* *0.004). Chicks exposed to low pattern richness were 3.63 times more likely to peck a feeder, regardless of color/pattern, than those exposed to high pattern richness (log hazard ratio for low pattern richness exposure = 1.291, *Z *=* *2.552, *p *=* *0.011, Figure 4, Supporting Information Figure S3). Chicks with higher spleen mass and higher testes asymmetry also had a much higher probability of pecking a feeder than less developed chicks (log hazard ratio for spleen mass = 7.771, *Z *=* *2.304, *p *=* *0.021; log hazard ratio for testes asymmetry = 3.916, *Z *=* *2.437, *p *=* *0.015, Figure 5, Supporting Information Figure S4). Body condition, body mass and tarsus length did not contribute to our final model of factors influencing predation.

## 4 DISCUSSION

The evolution of novel aposematic patterns in nature is a theme of intense debate among evolutionary biologists (Lindstrom, 1999; Mappes & Alatalo, 1997). If a novel aposematic pattern is not protected by previous predator education from similar warning patterns already extant in the region, the attention drawn to a bold, new pattern will subject it to a high degree of predator attack. Consequently, the intense predation on new patterns can slow or even inhibit their evolution (Turner, 1988), leaving scientists puzzled as to the selective mechanisms by which new patterns can evolve. Our initial expectation was that greater pattern diversity exposure would lead to greater hesitation time to attack imperfect phenotypes, as birds are expected to transfer knowledge of diverse visual cues to new prey (Svádová et al., 2009). Instead, we found that the effect of multiple aposematic models is dependent on the opportunity for social learning. Chicks exposed as a group to several patterns were less cautious than chickens exposed to one aposematic pattern. In contrast, when exposed individually, chickens are more cautious with a novel pattern when their previous aversive exposure involved multiple patterns.

### 4.1 Group exposure

Despite the low attack rate (food consumption) on aposematic feeders during the exposure phase, we found no evidence of discrimination between novel aposematic and brown prey during testing; whether previously exposed to low, medium or high color pattern training. This outcome suggests that novel imperfect mimics will not benefit from previous predator education on how to discriminate between edible and aposematic prey. Instead, all prey under low pattern richness benefit because socially trained predators are hesitant when facing any type of prey. In contrast, chicks exposed as a group to more than one aposematic pattern were less cautious and, thus, all prey patterns would be equally subjected to attack. This latter outcome has several possible causes. Young chickens may not be up to the cognitive task of integrating the many aposematic pattern features found in pattern‐rich environments. Similarly, because chicks needed to navigate both social interactions and multiple patterns during training sessions, they were distracted such that they were not conditioned to aposematic cues. Alternatively, chicks may have indeed learned to avoid specific aposematic phenotypes, but also eventually learned from sampling so many feeders that there was little consequence of testing new prey.

Our results suggest that social predators can encourage the evolution of imperfect mimicry in areas of low model pattern diversity as imperfect mimics receive a crucial time to escape a predation attempt. However, once multiple color patterns are established in a particular area, the information overload received by social predators can hinder the evolution of imperfect mimics as predators promptly attack their prey.

### 4.2 Individual exposure

As with the socially exposed subjects, individually exposed subjects did not discriminate against the novel aposematic feeder. However, individuals exposed to multiple patterns had a higher hesitation to feed from either feeder during their test trials. In pattern‐diverse areas, the uncertainty about the dangerousness of prey can make solitary predators more reluctant to try new food items presented to them. If so, in areas with many models and different aposematic patterns imperfect mimics are better protected because nonsocial predators will not immediately attack their prey, creating opportunity for escape.

Our individual subjects varied greatly in their latency to attack suggesting that motivational factors other than those caused by the treatments were at play. Difference in hunger is the most obvious explanation for this variation, but this seems unlikely given that chicks were fed ad libitum in their rearing brooder and each had equivalent opportunities to feed during the exposure events. Importantly, chick body condition did not explain latency to attack. Our results did, however, confirm our suspicion that the nutritional demands of alternative individual growth trajectories would contribute to explaining the variation in feeding hesitation by chicks. Although immune and reproductive development differs the most between strains of chickens, intrastrain differences among individuals in organ size or activity occur and can be found as early as day one (Apanius, 1998; de Reviers & Williams, 1984). Rapid growth of the spleen and development of adult‐like asymmetry in the testes were associated with greater urgency to begin feeding in our study, independent of body condition. This result suggests that individual organ growth trajectories may create feeding motivations that are not reflected by external morphological measurements, but affect the opportunity for the evolution of novel aposematic prey types. Individual variation on the willingness to attack, also documented in other species like the quail *Coturnix japonica* (Marples & Brakefield, 1995), can affect the evolution of new aposematic prey (Speed, 2000). When individuals with rapid development are more prone to attack aposematic prey, this can enhance the risk of extinction of new conspicuous prey. On the other hand, slow‐growing individuals could initially ease the selection on new aposematic prey.

Although we conclude that the individual variation in attack latency results from the motivation to feed imposed by the energetic demands of different growth trajectories, growth and learning are not independent; feeding successfully results both in an increase in body size and reinforces learning about how to feed effectively (English, Fawcett, Higginson, Trimmer, & Uller, 2016). Individuals with bold personalities often have a higher food intake rate (Biro & Stamps, 2008; Kurvers et al., 2010). Thus early differences in individual personality traits, such as boldness and the propensity to quickly explore space, may allow some chicks to begin feeding sooner and develop faster relative to individuals that are shy and slow to explore. Consequently the weaker aversion to the novel imperfect mimic by our more developed subjects may be the direct and independent result of the bold personality itself, rather than simply a product of the growth trajectory initiated by their precocity at feeding. We did not measure personality traits in our subjects, but in another bird, the great tit (*Parus major*), fast explorers showed shorter attack latency for an aposematic insect than slow individuals (Exnerová et al., 2010), a result similar to our chicks with advanced organ development. Nevertheless, the physiological demands of a bold personality may still be the driving force for the eagerness of such chicks to peck at aposematic prey. Bold individuals often have a higher metabolic rate than shy ones (Biro & Stamps, 2008), are at greater risk of starvation (Lichtenstein et al., 2017), and thus may need to be less catholic in their feeding, showing greater resistance to learning to avoid noxious prey (Exnerová et al., 2010). Clearly, the experimental disentanglement of predator personality, early development and motivation to feed discriminately is both relevant to our understanding of the evolution of mimicry and a complex challenge worthy of further research effort.

We demonstrated that color pattern diversity and social transmission of information might have an influence on the evolution of imperfect mimicry and mimicry in general, which corroborates mathematical models (Thorogood et al., 2017). However, we are aware that the evolution of imperfect mimicry may be facilitated by other extrinsic factors like niche preferences, predators with different visual systems (i.e., mammals vs. birds), and biogeographic history in areas with elevated model color diversity, as is the case for *Micrurus* in western Amazonia (Bosque et al., 2016). There are few cases where predation of coral snakes has been observed in nature (DuVal, Greene, & Manno, 2006) but it has been reported that in one specific site at least 90 species are potential predators of coral snakes (França, 2008). Predators of coral snakes have sufficient opportunity for social learning, given the number of species in a particular area (interspecific leaning) and the various degree of sociality of each species, ranging from less social species (red‐legged seriema *Cariama cristata*), to highly social species (greater ani, *Crotophaga major*).

Interestingly, this empirical demonstration of the effects of model diversity and social interaction lends some insight into how mimicry systems arise at all. In low model diversity systems, social predators facilitate the initial evolution of mimics while nonsocial predators are an opposing force. After a single color pattern model is established in a particular area, mediated by selection of social predators, the number of models/color patterns can further increase by selection of nonsocial predators (Figure 5). In this sense, in areas with high model color diversity, nonsocial predators will favor recently evolved mimics. Personal experience is probably more common than eavesdropped information, which might be another factor to explain why we find more mimics of coral snakes in areas of high color diversity of models (Davis Rabosky et al., 2016).

## 5 CONCLUSION

Newly evolved patterns can be favored by social learning in areas of low pattern diversity and disfavored by individual learning. These findings can shed light on the evolution of imperfect mimicry (Kikuchi & Pfennig, 2013), which were not previously explored. Our findings indicate that this phenomenon can be favored in areas of low and high model diversity by two distinct mechanisms. We suggest that imperfect mimicry can be favored in areas of high model diversity by reduced predation pressure as a result of attack hesitation by nonsocial predators. In areas of low pattern diversity, imperfect mimics can be better protected because social predators are not so cognitively overloaded that they become less prone to attack prey. Individual growth trajectory determines how predators will interact with their prey, making fast‐growing individuals less hesitant to attack. Our understanding of how information overload, growth trajectory, and the interrelationship between social and nonsocial predators on the evolution of imperfect mimicry will surely benefit from further consideration.

## CONFLICT OF INTERESTS

We have no competing interests.

## AUTHORS’ CONTRIBUTIONS

Renan Janke Bosque was responsible for the conception, design, acquisition, interpretation, and analysis and draft of the manuscript. J. P. Lawrence contributed to the acquisition of data, design, interpretation, and revision of the manuscript. Richard Buchholz contributed to the conception, design, interpretation, revision of the manuscript, and dissection of the chickens. Guarino Rinaldi Colli contributed to the conception, design, interpretation, analysis, and revision of the manuscript. Jessica Heppard aided with experimental design, data collection, and revision of the manuscript. Brice Noonan contributed to the conception, design, interpretation, and revision of the manuscript. All authors gave final approval for publication. All authors agreed to be accountable for all aspects of the work in ensuring that questions related to the accuracy or integrity of any part of the work are appropriately investigated and resolved.

## ETHICS STATEMENT

Approval granted to carry out the experiment IACUC 15‐009.

## DATA ACCESSIBILITY

No data deposition is applicable.

## References

[ece34272-bib-0001] Apanius, V. (1998). Ontogeny of immune function In Starck J. M., & Ricklefs R. E. (Eds.), Avian growth and development (pp. 203–222). New York, NY: Oxford University Press.

[ece34272-bib-0002] Aronsson, M. , & Gamberale‐Stille, G. (2012). Colour and pattern similarity in mimicry: Evidence for a hierarchical discriminative learning of different components. Animal Behaviour, 84, 881–887. 10.1016/j.anbehav.2012.07.011

[ece34272-bib-0003] Aubier, T. G. , & Sherratt, T. N. (2015). Diversity in Müllerian mimicry: The optimal predator sampling strategy explains both local and regional polymorphism in prey. Evolution, 69, 2831–2845. 10.1111/evo.12790 26456598

[ece34272-bib-0004] Bates, H. W. (1862). Contributions to an insect fauna of the Amazon Valley (Lepidoptera: Heliconidae). Transactions of the Linnean Society of London, 23, 495–566. 10.1111/j.1096-3642.1860.tb00146.x

[ece34272-bib-0005] Benjamini, Y. , & Yosef, H. (1995). Controlling the false discovery rate: A practical and powerful approach to multiple testing. Journal of the Royal Statistical Society, 57, 289–300.

[ece34272-bib-0006] Biro, P. A. , & Stamps, J. A. (2008). Are animal personality traits linked to life‐history productivity? Trends in Ecology & Evolution, 23, 361–368. 10.1016/j.tree.2008.04.003 18501468

[ece34272-bib-0007] Bocak, L. , & Yagi, T. (2010). Evolution of mimicry patterns in *Metriorrhynchus* (Coleoptera: Lycidae): The history of dispersal and speciation in Southeast Asia. Evolution, 64, 39–52. 10.1111/j.1558-5646.2009.00812.x 19674098

[ece34272-bib-0008] Bosque, R. J. , Noonan, B. P. , & Colli, G. R. (2016). Geographical coincidence and mimicry between harmless snakes (Colubridae: Oxyrhopus) and harmful models (Elapidae: Micrurus). Global Ecology and Biogeography, 25, 218–226. 10.1111/geb.12401

[ece34272-bib-0009] Brodie, E. D. (1993). Differential avoidance of coral snake banded patterns by free‐ranging avian predators in Costa Rica. Evolution, 47, 227–235. 10.1111/j.1558-5646.1993.tb01212.x 28568087

[ece34272-bib-0010] Brodie, E. D. , & Janzen, F. J. (1995). Experimental studies of coral snake mimicry: Generalized avoidance of ringed snake patterns by free‐ranging avian predators. Functional Ecology, 9, 186–190.

[ece34272-bib-0011] Brown, M. E. (1996). Assessing body condition in birds In Nolan V., & Ketterson E. D. (Eds.), Current ornithology (pp. 67–135). Boston, MA: Springer 10.1007/978-1-4615-5881-1

[ece34272-bib-0012] Buasso, C. M. , Leynaud, G. C. , & Cruz, F. B. (2006). Predation on snakes of Argentina: Effects of coloration and ring pattern on coral and false coral snakes. Studies on Neotropical Fauna and Environment, 41, 183–188. 10.1080/01650520600630725

[ece34272-bib-0013] Calhim, S. , & Montgomerie, R. (2015). Testis asymmetry in birds: The influences of sexual and natural selection. Journal of Avian Biology, 46, 175–185. 10.1111/jav.00503

[ece34272-bib-0014] Campbell, J. A. , & Lamar, W. W. (2004). The venomous reptiles of the Western Hemisphere. Volume 1. Ithaca, NY: Comstock Publishing Associates.

[ece34272-bib-0015] Davis Rabosky, A. R. , Cox, C. L. , Rabosky, D. L. , Title, P. O. , Holmes, I. A. , Feldman, A. , & McGuire, J. A. (2016). Coral snakes predict the evolution of mimicry across New World snakes. Nature Communications, 7, 11484 10.1038/ncomms11484 PMC4858746 27146100

[ece34272-bib-0016] de Reviers, M. , & Williams, J. B. (1984). Testis development and production of spermatozoa in the cockerel (*Gallus domesticus*) In Cunningham F. J., Lake P. E. & Hewitt D. (Eds.), Reproductive biology of poultry (pp. 183–202). Harlow, UK: British Poultry Science Ltd.

[ece34272-bib-0017] Drent, P. J. , Oers, K. V. , & Noordwijk, A. J. V. (2003). Realized heritability of personalities in the great tit (*Parus major*). Proceedings of the Royal Society B: Biological Sciences, 270, 45–51. 10.1098/rspb.2002.2168 12590770 PMC1691215

[ece34272-bib-0018] DuVal, E. H. , Greene, H. W. , & Manno, K. L. (2006). Laughing falcon (*Herpetotheres cachinnans*) predation on coral snakes (*Micrurus nigrocinctus*). Biotropica, 38, 566–568. 10.1111/j.1744-7429.2006.00162.x

[ece34272-bib-0019] Edmunds, M. (2000). Why are there good and poor mimics? Biological Journal of the Linnean Society, 70, 459–466. 10.1111/j.1095-8312.2000.tb01234.x

[ece34272-bib-0020] English, S. , Fawcett, T. W. , Higginson, A. D. , Trimmer, P. C. , & Uller, T. (2016). Adaptive use of information during growth can explain long‐term effects of early life experiences. The American Naturalist, 187, 620–632. 10.1086/685644 27104994

[ece34272-bib-0021] Exnerová, A. , Svádová, K. H. , Fučíková, E. , Drent, P. , & Štys, P. (2010). Personality matters: Individual variation in reactions of naive bird predators to aposematic prey. Proceedings of the Royal Society B: Biological Sciences, 277, 723–728. 10.1098/rspb.2009.1673 19889698 PMC2842751

[ece34272-bib-0022] França, F. G. R. (2008). O mimetismo das serpentes corais em ambientes campestres, savânicos e florestais da América do Sul. Tese de Doutorado. PhD, Universidade de Brasília.

[ece34272-bib-0023] Goerlich, V. C. , Nätt, D. , Elfwing, M. , Macdonald, B. , & Jensen, P. (2012). Transgenerational effects of early experience on behavioral, hormonal and gene expression responses to acute stress in the precocial chicken. Hormones and Behavior, 61, 711–718. 10.1016/j.yhbeh.2012.03.006 22465454

[ece34272-bib-0024] Greenberg, R. , & Mettke‐Hofmann, C. (2001). Ecological aspects of neophobia and neophilia in birds In Nolan V. Jr, & Thompson C. (Eds.), Current ornithology (pp. 119–178). New York, NY: Springer.

[ece34272-bib-0025] Ham, A. D. , Ihalainen, E. , Lindstrom, L. , & Mappes, J. (2006). Does colour matter? The importance of colour in avoidance learning, memorability and generalisation. Behavioral Ecology and Sociobiology, 60, 482–491. 10.1007/s00265-006-0190-4

[ece34272-bib-0026] Hart, N. S. (2001). The visual ecology of avian photoreceptors. Progress in Retinal and Eye Research, 20, 675–703. 10.1016/S1350-9462(01)00009-X 11470455

[ece34272-bib-0027] Hines, H. M. , & Williams, P. H. (2012). Mimetic colour pattern evolution in the highly polymorphic *Bombus trifasciatus* (Hymenoptera: Apidae) species complex and its comimics. Zoological Journal of the Linnean Society, 166, 805–826. 10.1111/j.1096-3642.2012.00861.x

[ece34272-bib-0028] Hinman, K. E. , Throop, H. L. , Adams, K. L. , Dake, A. J. , McLauchlan, K. K. , & McKone, M. J. (1997). Predation by free‐ranging birds on partial coral snake mimics: The importance of ring width and color. Evolution, 51, 1011–1014. 10.1111/j.1558-5646.1997.tb03684.x 28568580

[ece34272-bib-0029] Hotová Svádová, K. , Exnerová, A. , Kopečková, M. , & Štys, P. (2013). How do predators learn to recognize a mimetic complex: Experiments with naive Great Tits and aposematic Heteroptera. Ethology, 119, 814–830. 10.1111/eth.12121

[ece34272-bib-0030] Jiggins, C. D. , Mallarino, R. , Willmott, K. R. , & Bermingham, E. (2006). The phylogenetic pattern of speciation and wing pattern change in neotropical Ithomia butterflies (Lepidoptera: Nymphalidae). Evolution, 60, 1454–1466. 10.1111/j.0014-3820.2006.tb01224.x 16929662

[ece34272-bib-0031] John, J. L. (1994). The avian spleen: A neglected organ. Quarterly Review of Biology, 69, 327–351. 10.1086/418649 7972679

[ece34272-bib-0032] Kassambara, A. , Kosinski, M. , Biecek, P. , & Fabian, S. (2018). survminer: drawing survival curves using ‘ggplot2’. R package version 0.4.2. https://CRAN.R-project.org/package=survminer.

[ece34272-bib-0033] Kazemi, B. , Gamberale‐Stille, G. , Tullberg, Birgitta S. , & Leimar, O. (2014). Stimulus salience as an explanation for imperfect mimicry. Current Biology, 24, 965–969. 10.1016/j.cub.2014.02.061 24726157

[ece34272-bib-0034] Kikuchi, D. W. , & Pfennig, D. W. (2010). Predator cognition permits imperfect coral snake mimicry. The American Naturalist, 176, 830–834. 10.1086/657041 20950143

[ece34272-bib-0035] Kikuchi, D. W. , & Pfennig, D. W. (2013). Imperfect mimicry and the limits of natural selection. Quarterly Review of Biology, 88, 297–315. 10.1086/673758 24552099

[ece34272-bib-0036] Kraemer, A. C. , & Adams, D. C. (2014). Predator perception of Batesian mimicry and conspicuousness in a salamander. Evolution, 68, 1197–1206. 10.1111/evo.12325 24274647

[ece34272-bib-0037] Kurvers, R. H. J. M. , Prins, H. H. T. , van Wieren, S. E. , van Oers, K. , Nolet, B. A. , & Ydenberg, R. C. (2010). The effect of personality on social foraging: Shy barnacle geese scrounge more. Proceedings of the Royal Society B: Biological Sciences, 277, 601–608. 10.1098/rspb.2009.1474 19864281 PMC2842682

[ece34272-bib-0038] Lichtenstein, J. L. L. , Wright, C. M. , Luscuskie, L. P. , Montgomery, G. A. , Pinter‐Wollman, N. , & Pruitt, J. N. (2017). Participation in cooperative prey capture and the benefits gained from it are associated with individual personality. Current Zoology, 63, 561–567. 29033979 10.1093/cz/zow097 PMC5637736

[ece34272-bib-0039] Lindstrom, L. (1999). Experimental approaches to studying the initial evolution of conspicuous aposematic signalling. Evolutionary Ecology, 13, 605–618. 10.1023/A:1011004129607

[ece34272-bib-0040] Lindstrom, L. , Alatalo, R. V. , & Mappes, J. (1997). Imperfect Batesian mimicry ‐ The effects of the frequency and the distastefulness of the model. Proceedings of the Royal Society B: Biological Sciences, 264, 149–153. 10.1098/rspb.1997.0022

[ece34272-bib-0041] Lindstrom, L. , Alatalo, R. V. , & Mappes, J. (1999). Reactions of hand‐reared and wild‐caught predators toward warningly colored, gregarious, and conspicuous prey. Behavioral Ecology, 10, 317–322. 10.1093/beheco/10.3.317

[ece34272-bib-0042] Mappes, J. , & Alatalo, R. V. (1997). Batesian mimicry and signal accuracy. Evolution, 51, 2050–2053. 10.1111/j.1558-5646.1997.tb05129.x 28565127

[ece34272-bib-0043] Marples, N. M. , & Brakefield, P. M. (1995). Genetic variation for the rate of recruitment of novel insect prey into the diet of a bird. Biological Journal of the Linnean Society, 55, 17–27. 10.1016/0024-4066(95)90026-8

[ece34272-bib-0044] Møller, A. P. (1994). Directional selection on directional asymmetry: Testes size and secondary sexual characters in birds. Proceedings of the Royal Society B: Biological Sciences, 258, 147–151. 10.1098/rspb.1994.0155

[ece34272-bib-0045] Moore, D. F. (2016). Applied survival analysis using R, 1st ed. Basel, Switzerland: Springer International Publishing 10.1007/978-3-319-31245-3

[ece34272-bib-0046] Pekar, S. , Jarab, M. , Fromhage, L. , & Herberstein, M. E. (2011). Is the evolution of inaccurate mimicry a result of selection by a suite of predators? A case study using myrmecomorphic spiders. The American Naturalist, 178, 124–134. 10.1086/660287 21670583

[ece34272-bib-0047] R Core Team (2017). R: A language and environment for statistical computing. Vienna, Austria: R Foundation for Statistical Computing.

[ece34272-bib-0048] Rowe, C. , & Guilford, T. (2000). Aposematism: To be red or dead. Trends in Ecology & Evolution, 15, 261–262. 10.1016/S0169-5347(00)01897-8

[ece34272-bib-0049] Roze, J. A. (1996). Coral snakes of the Americas: Biology, identification, and venoms. Malabar, FL: Krieger Publishing Company.

[ece34272-bib-0050] Ruxton, G. D. , Sherratt, T. N. , & Speed, M. P. (2004). Avoiding attack: The evolutionary ecology of crypsis, warning signals and mimicry. Oxford, UK: Oxford University Press 10.1093/acprof:oso/9780198528609.001.0001

[ece34272-bib-0051] Savage, J. M. , & Slowinski, J. B. (1992). The colouration of the venomous coral snakes (family Elapidae) and their mimics (families Aniliidae and Colubridae). Biological Journal of the Linnean Society, 45, 235–254. 10.1111/j.1095-8312.1992.tb00642.x

[ece34272-bib-0052] Sherratt, T. N. (2002). The evolution of imperfect mimicry. Behavioral Ecology, 13, 821–826. 10.1093/beheco/13.6.821

[ece34272-bib-0053] Sherwin, C. M. , Heyes, C. M. , & Nicol, C. J. (2002). Social learning influences the preferences of domestic hens for novel food. Animal Behaviour, 63, 933–942. 10.1006/anbe.2002.2000

[ece34272-bib-0054] Skelhorn, J. , & Ruxton, G. D. (2010). Mimicking multiple models: Polyphenetic masqueraders gain additional benefits from crypsis. Behavioral Ecology, 22, 60–65.

[ece34272-bib-0055] Slagsvold, T. , & Wiebe, K. L. (2011). Social learning in birds and its role in shaping a foraging niche. Philosophical Transactions of the Royal Society B: Biological Sciences, 366, 969–977. 10.1098/rstb.2010.0343 PMC3049099 21357219

[ece34272-bib-0056] Smith, S. M. (1976). Predatory behaviour of young turquoise‐browed motmots, *Eumomota superciliosa* . Behaviour, 56, 309–320. 10.1163/156853976X00082

[ece34272-bib-0057] Speed, M. P. (2000). Warning signals, receiver psychology and predator memory. Animal Behaviour, 60, 269–278. 10.1006/anbe.2000.1430 11007635

[ece34272-bib-0058] Svádová, K. , Exnerová, A. , Štys, P. , Landová, E. , Valenta, J. , Fučíková, A. , & Socha, R. (2009). Role of different colours of aposematic insects in learning, memory and generalization of naïve bird predators. Animal Behaviour, 77, 327–336. 10.1016/j.anbehav.2008.09.034

[ece34272-bib-0059] Symula, R. , Schulte, R. , & Summers, K. (2001). Molecular phylogenetic evidence for a mimetic radiation in Peruvian poison frogs supports a Müllerian mimicry hypothesis. Proceedings of the Royal Society B: Biological Sciences, 268, 2415–2421. 10.1098/rspb.2001.1812 11747559 PMC1088895

[ece34272-bib-0060] Therneau, T. M. (2015). A package for survival analysis in S. version 2.38, <URL: https://CRAN.R-project.org/package=survival.

[ece34272-bib-0061] Thorogood, R. , Kokko, H. , & Mappes, J. (2017). Social transmission of avoidance among predators facilitates the spread of novel prey. Nature Ecology & Evolution, 2, 254–261. 29255302 10.1038/s41559-017-0418-x

[ece34272-bib-0062] Turner, J. R. G. (1988). The evolution of mimicry: A solution to the problem of punctuated equilibrium. The American Naturalist, 131, S42–S66.

