# Supplementary material for: Diversity of warning signal and social interaction influences the evolution of imperfect mimicry
Source: Ecol Evol. 2018 Jul 3;8(15):7490–9. doi: 10.1002/ece3.4272 (PMC6106177; doi:10.1002/ece3.4272)
Supplement: Supplementary file 1 [file ECE3-8-7490-s001.pdf]

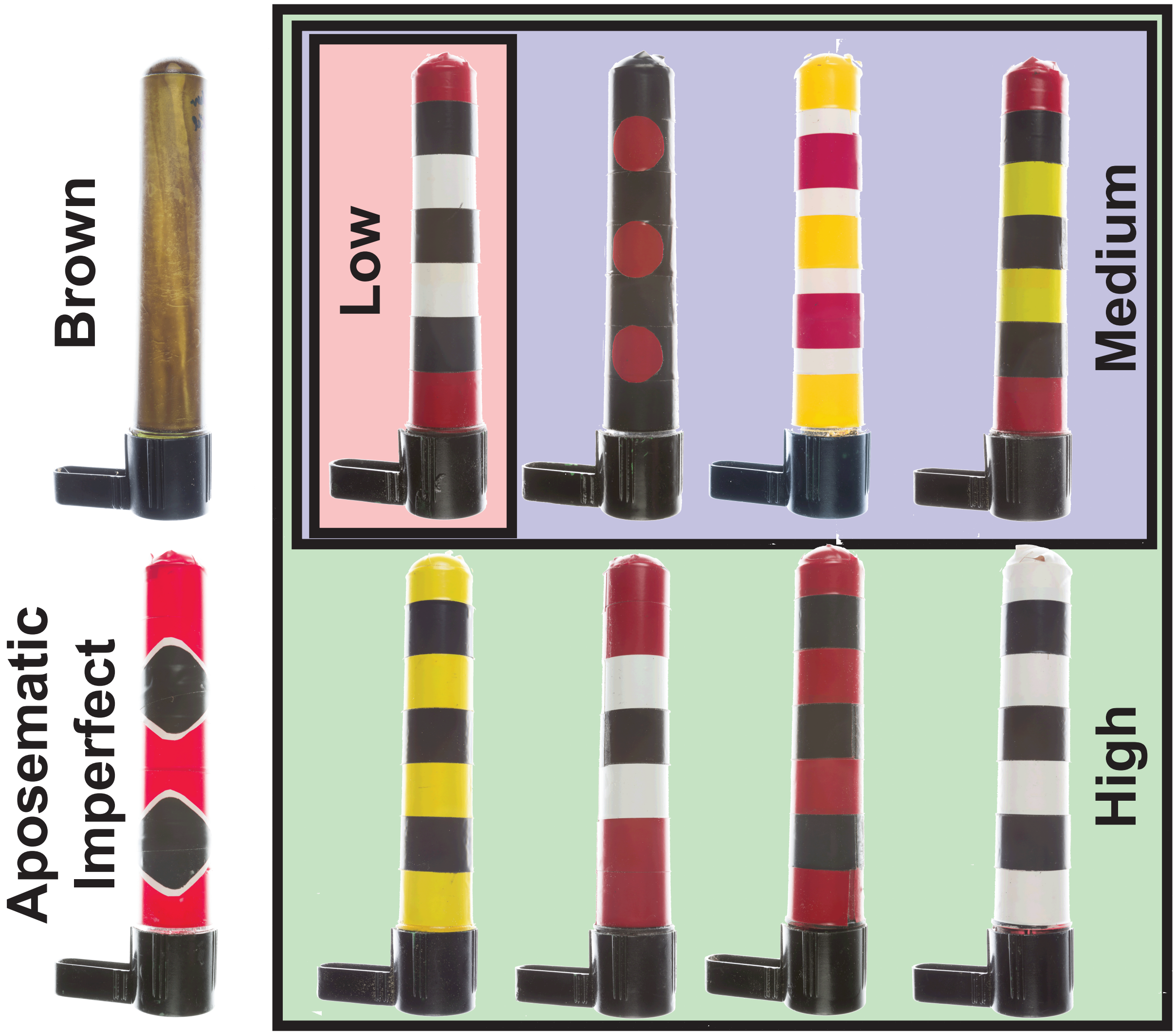

Supporting information 1. Bird feeders used during the experiment. Colours of the feeders are based on colouration of coral snakes of the genus *Micrurus* (model) and its mimic *Oxyrhopus rhombifer* (aposematic-imperfect). High, medium and low represent the number of colour pattern richness that birds were exposed prior to test with a brown or/and aposematic-imperfect feeder.
