# Supplementary material for: Diversity of warning signal and social interaction influences the evolution of imperfect mimicry
Source: Ecol Evol. 2018 Jul 3;8(15):7490–9. doi: 10.1002/ece3.4272 (PMC6106177; doi:10.1002/ece3.4272)
Supplement: Supplementary file 2 [file ECE3-8-7490-s002.pdf]

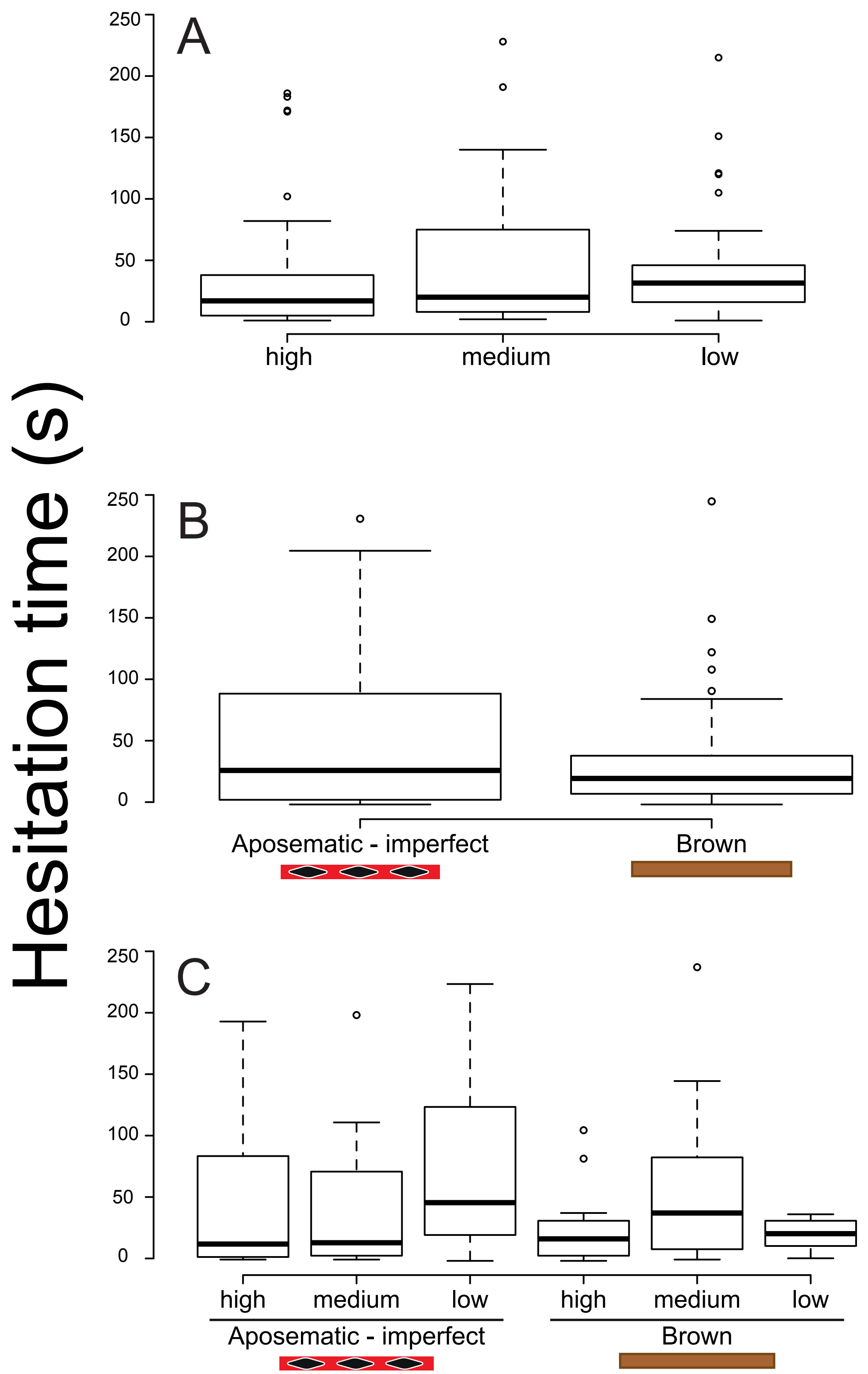

Supporting information 2. Hesitation time for chicks exposed as a group to different coral snake pattern richness to peck on feeders painted with non-aposematic (brown) or aposematic-imperfect patterns. A – hesitation time comparing colour pattern richness. B - hesitation time comparing non-aposematic imperfect versus brown feeder. C hesitation time comparing A & B.
